# High amniotic fluid fractalkine and MIP-1β levels are associated with intrauterine growth restriction: a prospective cohort study

**DOI:** 10.55730/1300-0144.5789

**Published:** 2023-12-16

**Authors:** Şehmus PALA, Remzi ATILGAN, Nevin İLHAN

**Affiliations:** 1 Department of Obstetrics and Gynecology, Fırat University School of Medicine, Elazığ, Turkiye; 2 Department of Biochemistry, Fırat University School of Medicine, Elazığ, Turkiye

**Keywords:** IUGR, fractalkine, MIP-1β, HIF-1α, amniotic fluid, inflammation

## Abstract

**Background/aim:**

Proinflammatory chemokines have been shown to play crucial roles in implantation, spiral artery invasion, and the fetomaternal immunological response. In this context, we investigated the levels of fractalkine (CX3CL1) and chemokine CC motif ligand 4 (CCL4 or MIP-1β) in maternal serum and amniotic fluids in pregnant women with intrauterine growth restriction (IUGR).

**Materials and methods:**

This prospective cohort study was carried out at Fırat University Obstetrics Clinic between January 1, 2022 and July 1, 2022. Group (G) 1: The control group consisted of 40 pregnant women who underwent elective cesarean section (CS) at 38–40 weeks of gestation. G2: A total of 40 pregnant women with IUGR at 28–37 weeks of gestation were included in the study group. Levels of tumor necrosis factor-alpha (TNF-α), interleukin-1 beta (IL-1β), interferon-gamma (IFN-γ), hypoxia-inducible factor-1 alpha (HIF-1α), macrophage inflammatory protein-1 beta (MIP-1β), and fractalkine were measured in maternal serum and amniotic fluid samples obtained during CS.

**Results:**

When maternal age was compared, no statistically significant difference was observed between G1 and G2 (p = 0.374). The number of gravidity was found to be statistically higher in G1 compared to G2 (p = 0.003). The mean gestational week was statistically higher in G1 (p < 0.001). Maternal serum MIP-1β (p = 0.03) and IFN-γ (p = 0.006) levels were higher in G1. The birth weight of the baby (p < 0.001) and umbilical cord blood gas pH value (p < 0.001) at birth were higher in G1. HIF-1α (p < 0.001), fractalkine (p < 0.001), MIP-1β (p < 0.001), TNF-α (p = 0.007), IL-1β (p < 0.001), and IFN-γ levels (p = 0.007) in amniotic fluid were higher in G2.

**Conclusion:**

Elevated levels of proinflammatory factors, including fractalkine and MIP-1β, along with inflammatory factors such as TNF-α, IL-1β, and IFN-γ, as well as increased HIF-1α levels in amniotic fluid, are associated with intrauterine growth restriction (IUGR) attributed to a hypoxic amniotic environment.

## 1. Introduction

While the diagnostic criteria for intrauterine growth restriction (IUGR) can vary widely based on national or international guidelines, a common simple definition involves abdominal circumference (AC) or estimated fetal weight (EFW) falling below the 10th percentile [1]. Gordjin et al. [2] proposed considering fetal growth rate alongside Doppler changes for impact IUGR diagnosis. The choice of biometric and Doppler reference intervals has been noted to vary significantly between countries, influencing the reported prevalence of IUGR and, consequently, impacting clinical management [3].

The etiology of most cases of IUGR not associated with fetal congenital malformations, genetic anomalies, or infectious causes is thought to stem from disruptions in uteroplacental circulation. Normal fetal growth necessitates adequate uteroplacental vascular dilation and adequate placental development. However, insufficient remodeling of spiral arteries has been reported as a predisposing factor for IUGR [4]. Inadequate remodeling results in the accumulation of foam cells in spiral arteries, narrowing of the lumen, and an increased susceptibility to acute atherosclerotic changes. These changes in the distal part of the connecting segment induce ischemia-reperfusion injury, severely restricting placental blood flow, and potentially leading to adverse obstetric outcomes [5].

Studies have demonstrated that vasculopathies arising during pregnancy can lead to embryonic malformations due to insufficient nutrient transport. In this context, the early presence of macrophages, pivotal in the development of vasculopathy, in the yolk sac in the early stages of pregnancy plays a crucial role in maintaining homeostasis and the functioning of structures that nourish the embryo. Disruptions in this process may result in abortion, intrauterine growth restriction, hypertension (HT), increased calcification in the placenta, preeclampsia (PE), and postnatal cardiovascular diseases [6].

Inflammatory mediator profiles can change even during normal pregnancy [7]. Throughout normal pregnancy, there is a shift from T-helper (T)1’ cells to T2 in the maternal immune system, leading to maternal immune tolerance and suppression [8]. In pregnancy complications such as recurrent spontaneous abortion and PE, there is an elevated presence of T1 cytokines, including interleukin (IL)-6, IL-8, interferon-gamma (IFN-γ), and tumor necrosis factor-alpha (TNF-α) [9–12]. As a result, these cytokines can be considered potential markers for high-risk pregnancies [13]. Pregnancy, therefore, is characterized as an inflammatory process, with an increase in maternal leukocyte activity and proinflammatory cytokines in the body [14,15]. It has been reported that the levels of placental proinflammatory cytokines are elevated in cases of IUGR, a condition largely associated with placental inflammation [16].

Chemokine ligand 1 (CX3CL1), now called fractalkine, stands as the sole identified member of the δ-chemokine family [17]. IL–2 causes upregulation of fractalkine receptor-1 (CX3CR1), inducing differentiation into CD4^+^ and CD8^+^ T cells. However, interferon-gamma (IFN-γ), TNF-α, and IL-1β, recognized as proinflammatory cytokines within blood vessels, stimulate the expression of fractalkine [18,19]. These characteristics endow fractalkine with significant roles in the immune response. Beyond its involvement in the regulation of the immune response, fractalkine is upregulated in processes like angiogenesis, hypoxic and inflammatory conditions [20]. Fractalkine, along with other cytokines such as the chemokine CC motif ligand 4 (CCL4 or MIP-1β), contributes to processes such as implantation, placental angiogenesis, trophoblast invasion into spiral arteries, responses to inflammatory and immunological factors at the uterine-placental interface, and the initiation of labor [21,22].

Understanding the mechanisms that govern immune balance during a typical pregnancy may provide insights into the immune profile in cases of IUGR [23]. Thus, our study aims to explore the levels of fractalkine and macrophage inflammatory protein-1 beta (MIP-1β or CCL4), a proinflammatory cytokine, in both maternal serum and amniotic fluid in IUGR cases.

## 2. Materials and methods

Our study included a total of 348 patients at 28–40 weeks of gestation who underwent cesarean section (CS) at Fırat University Faculty of Medicine Hospital Gynecology and Obstetrics Clinic between January 1, 2022 and July 1, 2022. Following the 2021 ACOG guideline [1], the study group comprised cases diagnosed with IUGR at 28–37 weeks of gestation. Patients who received betamethasone for lung maturation and underwent CS at least 48 h after the treatment were included in the study. The study was planned to be completed in 6 months, during which 348 cases were delivered via CS. Among these, 67 patients opted for elective CS at 38–40 weeks of gestation without any complications. The control group was randomly selected from these cases. Out of the 348 cases, 84 were diagnosed with IUGR. Among them, 44 had pregnancies complicated by other obstetric problems. Of these cases, 26 cases were complicated with PE, 9 cases with premature membrane rupture, 7 cases with gestational HT, 1 case with HELLP syndrome and 1 case with gestational cholestasis. These cases were excluded from the study. Thus, 40 IUGR patients whose pregnancies were not complicated by any disease, and who underwent elective CS constituted the study group (Figure).

With a total of 40 cases in our IUGR group, we matched the control group, resulting in the completion of the study with a total of 80 patients. Group 1 (n = 40) included patients with healthy pregnancies at 38–40 weeks, constituting the control group. Group 2 (n = 40) comprised patients diagnosed with IUGR during pregnancies at 28–37 weeks without any additional complications. Ultrasonographic and demographic data for all patients were recorded and evaluated prospectively. Informed consent forms were obtained from all pregnant women participating in the study. The study was carried out with the approval of the Fırat University Non-invasive Research Ethics Committee, dated 2021 and numbered 13–47. All patients provided informed consent, and the study was conducted in accordance with the principles of the Helsinki Declaration.

The study excluded cases involving fetal congenital and chromosomal anomalies, infections, premature rupture of membranes, multiple pregnancies, chronic maternal diseases, smoking during pregnancy, and the use of drugs that could impact serum enzyme levels. Additionally, uterine anomalies that may affect intrauterine development, such as HT, PE, HELLP syndrome, gestational diabetes, intrahepatic cholestasis of pregnancy, chronic organ failure, and pregnancies involving organ transplantation, were also not considered for inclusion. The control group consisted of third-trimester pregnant women who neither used drugs nor smoked. Cases with an estimated fetal weight below the 10th percentile for gestational age were defined as IUGR [1].

In our study, all pregnant women underwent CS based on obstetric indications. Following the baby’s birth, the umbilical cord was clamped before the first breath, and without separating the placenta. Subsequently, the umbilical artery was identified, and approximately 3 mL of blood was drawn into a heparinized syringe. The collected blood samples were immediately evaluated using a blood gas device. Acidosis was considered present if the pH was below 7.20.

### 2.1. IUGR diagnostic criteria

Pregnant women with abdominal circumference (AC) or estimated fetal weight (EFW) below the 10th percentile for gestational age were classified as having IUGR [1]. Additionally, Doppler evaluations of the umbilical artery (UA) and middle cerebral artery (MCA) were conducted on all participants in the study. Transabdominal color Doppler ultrasound (GE HealthCare Voluson E6 ultrasound system. Probe 4–8 D, Frequency 2–8 MHz/Austria) was employed to visualize the UA and MCA. After obtaining images of both vessels, flow impedance was assessed using pulsed-wave Doppler, and the pulsatility index (PI) was measured when three consecutive similar waveforms were obtained [24].

### 2.2. Obtaining a maternal blood and amniotic sample

Maternal serum and amniotic fluid samples were collected during CS. For serum analysis, approximately 5 cc of peripheral blood was collected from the forearm antecubital vein during delivery, following disinfection with an alcohol swab. The blood was then placed in a gel tube for serum analysis. After allowing the samples to coagulate at room temperature, they were centrifuged for 10 min at 4000 rpm/min within 15 min at the latest. The resulting serum was transferred into Eppendorf tubes and stored at −80 °C. Samples showing hemolysis or a lipemic appearance were excluded from the analysis. During CS, a small incision was made in the amniotic membrane using a scalpel, enabling a sufficient amount of amniotic fluid to drain into a small, sterile container. Subsequently, around 10 mL of amniotic fluid was drawn from this container into a syringe. Special care was taken during this process to minimize the mixing of maternal blood with amniotic fluid. After the fetus was delivered, and the umbilical cord was clamped, any cases with cloudiness observed in the amnion sample were excluded from the study, as it suggested a potential high contamination with maternal blood. Amniotic fluid from each case was collected under sterile conditions and stored at −80 °C until further analysis. All samples were processed in the Biochemistry Laboratory of Fırat University, Faculty of Medicine.

### 2.3. Biochemical measurements

TNF-α, IL-1β, IFN-γ, HIF-1α, MIP-1β, and fractalkine levels in both maternal serum and amniotic fluid were measured using the human Enzyme–Linked Immunosorbent Assay (ELISA) method. All biochemical measurements were conducted following the respective kit procedures. The city, country, company, measurement range, and sensitivity of the ELISA kits used are shown in detail in Table 1.

Absorbances were spectrophotometrically read at 450 nm using an EPOCH 2 microplate reader (Bio Tek Instrument, Inc, USA) to obtain the results.

### 2.4. Statistical analysis

All statistical analyses were conducted using the SPSS 22.0 package program (IBM Corporation, Armonk, NY, USA). The Shapiro–Wilk test was employed to assess whether numerical variables followed a normal distribution. In cases where numerical variables were not normally distributed, the Mann–Whitney U test was used for comparisons between the two groups. Relationships between categorical variables were examined using the chi–squared test. Descriptive statistics for numerical variables were presented as mean and standard deviation, while categorical variables were described using numbers and percentages. A significance level of p < 0.05 was considered statistically significant.

## 3. Results

### 3.1. Comparison of maternal demographic parameters

The average maternal age was 29.5 years (range: 24–40) in G1 and 28 years (range: 19–39) in G2. When comparing both groups, no statistically significant difference was observed (p = 0.374). The average number of gravidity was statistically higher in G1 compared to G2 [4 (range: 1–8) versus 2 (range: 1–9)], (p = 0.003). The average parity number was also statistically higher in G1 compared to G2 [2 (range: 0–4) versus 0.5 (range: 0–5)], (p = 0.002). Moreover, the mean gestational week was statistically higher in G1 compared to G2 [39 (range: 38–40) versus 35 (range: 28–37)], (p < 0.001), (Table 2).

### 3.2. Comparison of maternal serum biochemical parameters

The mean HIF-1α levels in maternal serum were 2.43 ng/mL (range: 1.18–4.13) in G1 and 1.92 ng/mL (range: 1.18–8.46) in G2. When both groups were compared, no statistically significant difference was observed (p = 0.149). The mean serum fractalkine levels were 0.28 ng/mL (range: 0.23–37) in G1 and 3 ng/mL (range: 0–91.8) in G2, with no statistically significant difference between the groups (p = 0.161). However, mean serum MIP-1β levels were statistically higher in G1 compared to G2 [24 pg/mL (range: 1.24–139) versus 14.7 pg/mL (range: 0–181.1)], (p = 0.030). Mean serum TNF-α levels were measured as 107 ng/L (range: 7.3–380) in G1 and 85 ng/L (range: 0–65121) in G2. When both groups were compared, no statistically significant difference was observed (p = 0.488). The mean serum IL-1β levels were 1171 pg/mL (range: 72–634543) in G1 and 642 pg/mL (range: 0–76272) in G2. Although no statistically significant difference was observed (p = 0.055), mean serum IFN-γ levels were significantly higher in G1 compared to G2 [104 ng/L (range: 1.87–300) versus 73 ng/L (range: 0–529)], (p = 0.006), (Table 2).

### 3.3. Comparison of fetal parameters

The mean fetal weight was significantly higher in G1 compared to G2 [3200 g (range: 2600–4100) versus 1600 g (range: 620–2710)], (p < 0.001). The mean 1st minute APGAR score was also significantly higher in G1 than in G2 [8 (range: 7–9) versus 6 (range: 6–7)], (p < 0.001). Likewise, the average 5th minute APGAR score was significantly higher in G1 than in G2 [9 (range: 9–10) versus 8 (range: 7–10)], (p < 0.001). Moreover, the mean umbilical cord blood gas pH value was higher in G1 than in G2 [7.34 (range: 7.29–7.42) versus 7.3 (range: 6.98–7.4)], (p < 0.001), (Table 3).

### 3.4. Comparison of biochemical parameters in amniotic fluid

Mean HIF-1α levels were significantly higher in G2 compared to G1 [2.13 ng/mL (range: 1.12–2.72) versus 3.16 ng/mL (range: 0.12–15.3)], (p < 0.001). Mean fractalkine levels were also significantly higher in G2 compared to G1 [0.25 ng/mL (range: 0.23–2.91) versus 3.7 ng/mL (range: 0.25–25)], (p < 0.001). Mean MIP-1β levels were significantly higher in G2 compared to G1 [10.8 pg/mL (range: 4.9–22.9) versus 31.9 pg/mL (range: 8.4–52.2)], (p < 0.001). Additionally, mean TNF-α levels were significantly higher in G2 compared to G1 [79 ng/mL (range: 20–102) versus 113 ng/mL (range: 7.5–265)], (p = 0.007). Mean IL-1β levels were significantly higher in G2 compared to G1 [625 pg/mL (range: 33.8–1290) versus 1078 pg/mL (range: 76–3891)], (p < 0.001). Finally, mean IFN-γ levels were significantly higher in G2 compared to G1 [21 ng/mL (range: 16–66) versus 97.8 ng/mL (range: 29.1–226.9)], (p < 0.001), (Table 3).

### 3.5. Comparison of MCA and umbilical artery Doppler flow parameters

The average MCA–PI value was measured as 1.41 (range: 1.15–2.29) in G1 and 1.478 (range: 0.76–2.8) in G2. When both groups were compared, no statistically significant difference was observed (p = 0.298). Similarly, the mean MCA–SD (MCA–systolic-diastolic) value was measured as 4.08 (range: 2.98–7.79) in G1 and 3.99 (range: 2.77–10.3) in G2, with no statistically significant difference between the groups (p = 0.644). The average UA–PI value was measured as 1.06 (range: 0.64–1.24) in G1 and 1.06 (range: 0.7–1.63) in G2, and no statistically significant difference was observed when both groups were compared (p = 0.114). The mean UA–SD (UA–systolic-diastolic) value was measured as 3 (range: 1.95–3.5) in G1 and 3.05 (range: 2.22–4.5) in G2, and again, no statistically significant difference was observed between the groups (p = 0.097), (Table 4).

## 4. Discussion

In our study, we observed higher levels of MIP-1β and IFN-γ in maternal serum in the control group, whereas they were elevated in amniotic fluid in the IUGR group. However, we demonstrated that in cases of IUGR, HIF-1α, fractalkine, TNF-α, and IL-1β levels were higher only in amniotic fluid. Our results support that IUGR is associated with a hypoxic and inflammatory process. The significant increase in the level of fractalkine, a potent proinflammatory factor, particularly in amniotic fluid compared to the control group, may contribute to a better understanding of the immune profile in the pathogenesis of IUGR.

Additional monitoring parameters, such as Doppler velocity measurement, helps to determine the intrauterine fetal risk along with assessing impaired uteroplacental blood flow. These parameters offer additional benefits beyond the criterion of decreased fetal growth rate in cases of suspected IUGR. Redistribution of cerebral blood flow creates changes in Doppler flow, reflecting fetal hypoxemia. Detection of these changes contributes to the identification of IUGR and aids in predicting adverse fetal outcomes [25]. Therefore, in cases where assessing fetal growth alone may not be sufficient to identify IUGR, incorporating Doppler velocity measurement of the uterine, umbilical, and middle cerebral arteries proves to be valuable. Doppler velocity measurement allows for the evaluation of the entire spectrum of fetal vascular compensatory abnormalities and uteroplacental abnormalities throughout pregnancy. The Trial of Umbilical and Fetal Flow in Europe (TRUFFLE) group’s expert consensus recommends early fetal growth restriction (FGR) as fetal abdominal circumference (AC) <10% and umbilical artery pulsatility index (UA–PI) >95% [26]. In our study, we assessed possible fetal distress by incorporating UA and MCA Doppler measurements, in addition to the fetal biophysical profile, during the follow-up of all cases with IUGR. However, we found no significant differences between the UA and MCA Doppler PI and systolic-diastolic (SD) values in both our groups.One possible explanation is that we delivered our IUGR patients before the development of fetal distress findings in our study, possibly leading to similar Doppler parameters in both groups. It is essential to note that the gestational weeks did not precisely match between our control and IUGR groups. Additionally, patients in our IUGR group that developed fetal distress and were delivered were excluded from the study due to complications such as PE, HT, HELLP, and premature rupture of membranes (PROM). For these reasons, although we presented the PI and SD indices of the umbilical artery and MCA, we decided against performing any correlation analysis to avoid potentially misleading results.

Since IUGR, which is the second leading cause of fetal morbidity [27], has no known treatment, preterm delivery remains the only appropriate intervention. However, it is important to note that preterm birth may contribute to the development of insulin resistance, obesity, and certain chronic diseases in both childhood and adulthood [28–30]. In our IUGR cases, the gestational week and fetal birth weight at birth were significantly lower than in the control group. This suggests that IUGR is associated with early termination of pregnancy and lower fetal weight at birth.

Extravillous trophoblasts (EVTs) derived from human first-trimester blastocysts invade the decidua, comprising an immune cell population primarily composed of decidual natural killer (dNK) cells and macrophages [31], forming a high-efflux channel [32]. Inadequate invasion of EVT, however, leads to reduced uteroplacental blood flow due to insufficient remodeling of spiral arteries. This, in turn, results in placental hypoxia, causing the secretion of elevated levels of soluble antiangiogenic factors into the maternal serum. This antiangiogenic environment induces endothelial cell activation and dysfunction [33].

Pathological expression of HIF-1α in the placenta can result in inadequate remodeling of maternal spiral arteries, leading to impaired uteroplacental perfusion [34]. Failure to down-regulate HIF-1α after the first trimester [35], a period when placental oxygenation typically increases, leads to the accumulation of HIF-1α and, consequently, the development of PE and inflammation-induced IUGR [36].

In our study, we found that maternal serum HIF-1α levels were similar in both our control and IUGR groups, while they were significantly elevated in the amniotic fluid compared to the control group. Amniotic fluid (AF) comprises fetal and maternal compartments, fluids released from amniotic tissues, and fetal urine [37]. Therefore, considering that amniotic fluid may reflect fetal tissues and fluids, this suggests that the hypoxic amniotic environment in our IUGR cases may also be associated with a hypoxic fetus compared to normal pregnancy. The lower umbilical cord arterial blood pH values and lower APGAR scores obtained during delivery compared to normal fetuses also support the relationship between IUGR and hypoxia.

Maternal immunity plays a crucial role in regulating proinflammatory cytokines such as TNF-α, IL-1β, and IL–6 in the maternal-fetal system [38]. Reports indicate that TNF-α concentration is higher in IUGR cases with placental insufficiency compared to normal pregnancies [9]. Additionally, elevated TNF-α levels can activate IL-1β to protect cytotrophoblasts from the cytotoxic effects of TNF-α [39]. However, in cases of IUGR, an inflammatory response is induced due to increased transcription levels of proinflammatory cytokines [40]. Studies have shown that TNF-α, along with midtrimester amniotic fluid cytokines, is associated with IUGR [41]. It has also been demonstrated that maternal plasma protein levels are not associated with intraamniotic infection (IAI), and there is a weak correlation between plasma cytokine/chemokine levels and AF levels [42]. In a separate study, it was found that maternal serum protein levels have limited value in the noninvasive diagnosis of pregnancies with premature rupture of membranes [43]. In our study, we observed that TNF-α and IL-1β levels in the amniotic fluid were significantly increased in the IUGR group. However, maternal serum TNF-α and IL-1β levels were similar in both groups. This finding suggests that the inflammatory process in IUGR may progress more actively at the fetal interface rather than at the maternal interface.

IFN-γ plays a dominant role in regulating EVT invasion and recruiting peripheral natural killer (pNK) cells, in addition to the decidua [44]. In pregnant mice, IFN-γ secreted by dNK cells has been shown to play an important role in the remodeling of the spiral artery [45]. IFN-γ controls other cytokines during pregnancy and plays an important role in the formation and maintenance of maternal-fetal interactions. For the continuation of pregnancy to be possible, it is essential that trophoblasts are not rejected by maternal leukocytes. This is facilitated by the major histocompatibility complex through the induction of IFN-γ. However, it has been demonstrated that elevated levels of IFN-γ can lead to pregnancy loss [46].

In our study, we observed that the levels of IFN-γ in maternal serum were lower in our IUGR group than in the control group. This may reflect the complex dynamics of maternal-fetal immune interactions essential for maintaining pregnancy. Further research is needed to better understand this relationship. However, the higher levels of IFN-γ in the amniotic fluid in our IUGR group compared to the control group suggest a potential association between increased IFN-γ levels and IUGR. It has been demonstrated that betamethasone, a corticosteroid routinely administered to accelerate fetal lung maturation in pregnant women at risk of premature birth, can suppress inflammatory processes [47,48]. Studies have shown that betamethasone, administered in two doses of 12 mg/day with a 24-h interval in cases of threatened preterm labor, significantly reduces the endocervical concentration of IL-1β, TNF-α, and IFN-γ after 48 h. IL-1β, TNF-α, and IFN-γ are known to play roles in the etiology of preterm birth [49,50]. It is important to consider that the betamethasone treatment used in our IUGR cases may influence the levels of inflammatory processes in maternal serum. Therefore, the lower level of IFN-γ in maternal serum in our IUGR cases may be attributed to the betamethasone treatment.

The chemokine fractalkine is expressed in endothelial and smooth muscle cells under inflammatory conditions [51]. The fractalkine receptor (CX3CR1) is found in NK cells, monocytes, and CD8 + T lymphocytes. Fractalkine is responsible for the chemotaxis of leukocytes, NK cells, and T cells at the site of inflammation. Apart from chemotaxis, the primary function of fractalkine lies in regulating the immune system [52]. In the reproductive system, fractalkine is present on both the maternal and placental sides. Fractalkine, secreted from syncytiotrophoblasts and shed into the maternal circulation, constitutes the primary source of placental fractalkine. Upon activation of CX3CR1 in the placenta, fractalkine can induce angiogenesis through the two-step mechanism HIF-1α/VEGF and stimulate integrin-dependent trophoblast migration into the spiral arteries, a crucial step in the process of trophoblast invasion [53]. Hypoxia may independently stimulate both fractalkine synthesis and CX3CR1 expression in placental perfused lobules [54]. Alongside other cytokines such as fractalkine, MIP-1β is involved in implantation processes, trophoblast invasion into spiral arteries, placental angiogenesis, and responding to inflammatory and immunological factors at the uterus-placental interface, as well as the induction of labor [21,53]. MIP-1β plays a role in immune cell chemotactic activity [55], and it has been demonstrated to be involved in implantation [56]. Serum MIP-1β levels have been shown to increase in active infections during pregnancy [57]. In your study, maternal serum fractalkine levels were found to be similar between the IUGR and control groups, while there was a significant increase in amniotic fluid fractalkine levels in the IUGR group. This observation may suggest a connection between the increased HIF-1α levels in amniotic fluid and fractalkine. However, maternal serum MIP-1β levels were higher in the control group than in the IUGR group in our study. It is known that certain aspects of immunity are suppressed during pregnancy to allow maternal immune tolerance of fetal antigens. However, NK and T cells from pregnant women have been shown to exhibit enhanced IFN-γ and MIP-1β responses to influenza A virus compared to nonpregnant women [58]. The higher maternal serum MIP-1β levels in our control group compared to the IUGR group may be attributed to an undiagnosed subclinical infection. However, the higher MIP-1β levels in the amniotic fluid in the IUGR group compared to the control group may support the notion that fetuses with IUGR are exposed to an inflammatory environment. Further studies could shed light on this issue. The limited number of cases and the inability to examine the placentas biochemically and immunohistochemically represent the limitations of our study. Other limitations include not performing biochemical examinations on umbilical cord blood, nonmatching gestational weeks between the control and IUGR groups, and a lack of body mass index data. It should be considered that administering betamethasone in IUGR cases may also affect inflammatory parameters in maternal serum. Additionally, it should be noted that the placenta is an aging organ, and cytokine levels vary depending on the week of pregnancy.

In conclusion, elevated levels of fractalkine, MIP-1β, TNF-α, IL-1β, IFN-γ, and HIF-1α in amniotic fluid may contribute to an inflammatory and hypoxic amniotic environment, potentially associated with IUGR and adverse fetal outcomes. Understanding the mechanism involving amniotic fluid and/or placental fractalkine in fetuses with IUGR could be important for the development of new therapeutic strategies. An increased level of amniotic fluid fractalkine is associated with inflammation-induced IUGR.

## Figures and Tables

**Figure:**
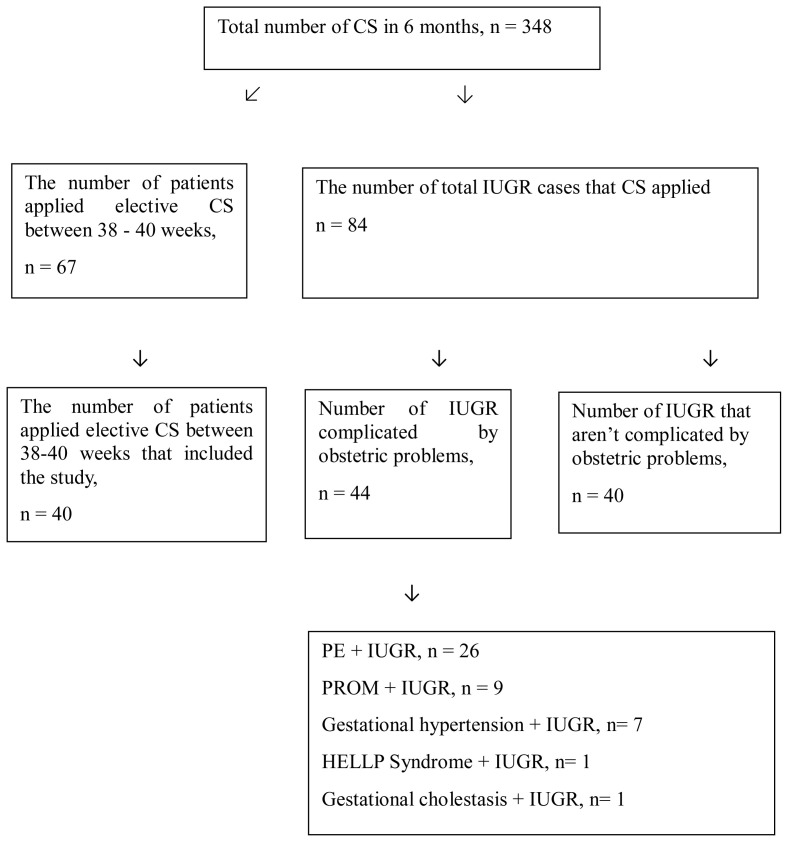
Diagram of patients according to inclusion and exclusion criteria.

**Table 1:** Country, company, catalog number, kit measurement range, and kit sensitivity of the enzyme-linked immunosorbent assay (ELISA) kits used in the study.

Parameters	Company, city and country	Catalog number	Measuring range	Sensitivity
TNF-α	Sunred Biotechnology Company, Shanghai, China	201–12–0083	3–900 (ng/L)	2.827 (ng/L)
IL-1β	Sunred Biotechnology Company, Shanghai, China	201–12–0144	200–800 (pg/mL)	15.013 (pg/mL)
IFN-γ	Sunred Biotechnology Company, Shanghai, China	201–12–0106	2–600 (ng/L)	1.706 (ng/L)
HIF-1α	Elabscience Biotechnology Company, Shanghai, China	E-EL–H1277	0.156–10 (ng/mL)	0.094 (ng/mL)
MIP-1β	Sunred Biotechnology Company, Shanghai, China	201–12–0088	0.5–150 (pg/mL)	0.432 (pg/mL)
Fractalkine	Sunred Biotechnology Company, Shanghai, China	201–12–2102	0.2–30 (ng/mL)	0.102 (ng/mL)

Abbreviations: TNF–α = tumor necrosis factor-alpha; IL-1β = interleukin-1 beta; IFN-γ = interferon-gamma; HIF-1α = hypoxia-inducible factor-1 alpha; MIP-1β = macrophage inflammatory protein-1 beta.

**Table 2:** Demographic parameters and HIF-1α, fractalkine, MIP-1β, TNF-α, IL-1β, IFN-γ, levels in maternal serum of G1 and G2, values are presented as median (minimum–maximum), p < 0.05 was considered to be statistically significant.

Parameters	G1 (n = 40)	G2 (n = 40)	p-values
Age (years)	29.5 (24–40)	28 (19–39)	0.374
Gravidity	4 (1–8)	2 (1–9)	0.003*
Parity	2 (0–4)	0.5 (0–5)	0.002*
Gestational week	39 (38–40)	35 (28–37)	<0.001*
HIF-1α, ng/mL	2.43 (1.18–4.13)	1.92 (1.18–8.46)	0.149
Fractalkine, ng/mL	0.28 (0.23–37)	3 (0–91.8)	0.161
MIP-1β, pg/mL	24 (1.24–139)	14.7 (0–181.1)	0.030*
TNF-α, ng/L	107 (7.3–380)	85 (0–65121)	0.488
IL-1β, pg/mL	1171 (72–634543)	642 (0–76272)	0.055
IFN-γ, ng/L	104 (1.87–300)	73 (0–529)	0.006*

Abbreviations: G1 = Control group; G2 = IUGR (Intrauterine growth restriction) group; HIF-1α = hypoxia-inducible factor-1 alpha; MIP-1β = macrophage inflammatory protein-1 beta; TNF-α = tumor necrosis factor-alpha, IL-1β = interleukin-1 beta, IFN-γ = interferon-gamma,

* = Compared with G1.

**Table 3:** The birth weight of the baby, Apgar scores in 1th and 5th minutes, blood gas pH value and HIF-1α, Fractalkine, MIP-1β, TNF-α, IL-1β, IFN-γ levels in amniotic fluid of G1 and G2, values are presented as median (minimum–maximum), p < 0.05 was considered to be statistically significant.

Parameters	G1 (n = 40)	G2 (n = 40)	p values
Baby weight (g)	3200 (2600–4100)	1600 (620–2710)	<0.001*
Apgar scores in 1th minute	8 (7–9)	6 (6–7)	<0.001*
Apgar scores in 5th minute	9 (9–10)	8 (7–10)	<0.001*
Blood gas pH value	7.34 (7.29–7.42)	7.3 (6.98–7.4)	<0.001*
HIF-1α, ng/mL	2.13 (1.12–2.72)	3.16 (0.12–15.3)	<0.001*
Fractalkine, ng/mL	0.25 (0.23–2.91)	3.7 (0.25–25)	<0.001*
MIP-1β, pg/mL	10.8 (4.9–22.9)	31.9 (8.4–52.2)	<0.001*
TNF-α, ng/L	79 (20–102)	113 (7.5–265)	0.007*
IL-1β, pg/mL	625 (33.8–1290)	1078 (76–3891)	<0.001*
IFN-γ, ng/L	21 (16–66)	97.8 (29.1–226.9)	<0.001*

Abbreviations: G1 = Control group; G2 = IUGR (Intrauterine growth restriction) group; HIF-1α = hypoxia-inducible factor-1 alpha; MIP-1β = macrophage inflammatory protein-1 beta; TNF-α = tumor necrosis factor-alpha, IL-1β = interleukin– 1 beta, IFN-γ = interferon-gamma,

* = Compared with G1.

**Table 4:** MCA–PI, MCA–SD, UA–PI, and UA–SD Doppler results obtained in both groups. Values are presented as median (minimum–maximum).

Parameters	G1 (n = 40)	G2 (n = 40)	p-values
MCA–PI	1.41 (1.15–2.29)	1.47800 (0.76–2.8)	0.298
MCA–SD	4.08 (2.98–7.79)	3.99 (2.77–10.3)	0.644
UA–PI	1.06 (0.64–1.24)	1.06 (0.7–1.63)	0.114
UA–SD	3 (1.95–3.5)	3.05 (2.22–4.5)	0.097

p < 0.05 was considered to be statistically significant.

Abbreviations: G1 = Control group; G2 = IUGR (Intrauterine growth restriction) group, MCA-PI = middle cerebral artery pulsatile index; MCA–SD = middle cerebral artery systolic-diastolic ratio; UA–PI = umbilical artery pulsatile index; UA–SD = umbilical artery systolic-diastolic ratio.
